# Health policy and systems research: building momentum and community

**DOI:** 10.2471/BLT.14.149393

**Published:** 2014-12-01

**Authors:** Abdul Ghaffar, Nhan Tran, John-Arne Røttingen, Marie-Paule Kieny

**Affiliations:** a Alliance for Health Policy and Systems Research, World Health Organization, avenue Appia 20, 1211 Geneva 27, Switzerland.; b Division of Infectious Disease Control, Norwegian Institute of Public Health, Oslo, Norway.; c Health Systems and Innovation Cluster, World Health Organization, Geneva, Switzerland.

In October 2014, nearly 2000 people from 125 countries shared and debated issues that are critical to improving the performance of health systems, at the Third Global Symposium on Health Systems Research, in Cape Town, South Africa.^1^ Such research was barely visible on the global health agenda until 1996, when the World Health Organization’s (WHO’s) Ad Hoc Committee on Health Research identified health systems research as an important but neglected field.^2^ Now, as shown by the success of three global symposia, governments worldwide clearly recognize the need for such research to build resilient health systems. A wide range of public health disasters – including the current Ebola epidemic – have drawn attention to the devastation that can rapidly develop in countries with weak health systems.

The three global symposia, WHO’s development of a strategy on health policy and systems research in 2012^3^ and the 2013 world health report on research for universal health coverage^4^ represent some important milestones in the field. Within the last decade, many collaborations and partnerships have emerged and dedicated entities – such as the Alliance for Health Policy and Systems Research and the professional society Health Systems Global – have been developed. The participation of the United States of America’s Global AIDS Coordinator in the Cape Town symposium is one indicator that health policy and systems research is valued by actors who have historically identified with specific disease areas. Such actors are now turning to systems sciences and applying the methods of health policy and systems research to overcome the common challenges of implementation, integration and sustainability.

Although political engagement will be critical to the strengthening of health systems, it must go beyond those decision-makers who act at national level. Most changes to health systems occur at subnational levels because it is district health officers and senior programme managers who are largely responsible for the implementation of national policies. Policy and systems research – including implementation research – must be able to respond to the needs of the system. The recently published *Statement on advancing implementation research and delivery science* called for greater engagement of implementers.^5^

While the health-systems landscape of today is a stark contrast to that of the 1980s, the resources available for health policy and systems research remain less than those available for many other areas of health research.^6^ There is a particular need for efficiency, through alignment, coordination and collaboration. Thematic working groups within the Health Systems Global organization, the use of tools such as the *Health policy and systems research: a methodology reader*,^7^ and its related implementation research guide,^8^ offer opportunities for learning and synergies. Exercises for setting research priorities – such as those led by the Alliance for Health Policy and Systems Research^9^ – can greatly enhance the impact of existing efforts and minimize duplication of work.

Now that the field has the long-sought attention of the global health community, the key actors need to demonstrate that they can deliver the potential that they have promised. The Alliance for Health Policy and Systems Research, Health Systems Global and WHO – bringing, respectively, a history of funding this research, broad membership and global convening power – are committed to working together to ensure that the full potential of health policy and systems research is realized. As the nature of the challenges experienced by health systems continues to evolve, so too will this field. All key actors will have to engage and collaborate to the fullest extent to ensure that health systems reap the maximum benefits of such research.
